# MicroRNA Signature in Human Normal and Tumoral Neural Stem Cells

**DOI:** 10.3390/ijms20174123

**Published:** 2019-08-23

**Authors:** Andrea Diana, Giuseppe Gaido, Daniela Murtas

**Affiliations:** 1 Department of Biomedical Sciences, University of Cagliari, 09042 Monserrato (Cagliari), Italy; 2 Department of Surgery, Cottolengo Mission Hospital Charia, 60200 Meru, Kenya

**Keywords:** human, embryonic stem cell, neural stem cell, pluripotent stem cell, miRNA, brain tumor, glioma stem cell, tumor suppressor miRNA, oncomiRNA

## Abstract

MicroRNAs, also called miRNAs or simply miR-, represent a unique class of non-coding RNAs that have gained exponential interest during recent years because of their determinant involvement in regulating the expression of several genes. Despite the increasing number of mature miRNAs recognized in the human species, only a limited proportion is engaged in the ontogeny of the central nervous system (CNS). miRNAs also play a pivotal role during the transition of normal neural stem cells (NSCs) into tumor-forming NSCs. More specifically, extensive studies have identified some shared miRNAs between NSCs and neural cancer stem cells (CSCs), namely miR-7, -124, -125, -181 and miR-9, -10, -130. In the context of NSCs, miRNAs are intercalated from embryonic stages throughout the differentiation pathway in order to achieve mature neuronal lineages. Within CSCs, under a different cellular context, miRNAs perform tumor suppressive or oncogenic functions that govern the homeostasis of brain tumors. This review will draw attention to the most characterizing studies dealing with miRNAs engaged in neurogenesis and in the tumoral neural stem cell context, offering the reader insight into the power of next generation miRNA-targeted therapies against brain malignances.

## 1. Introduction

### 1.1. miRNAs and Neurogenesis 

From the beginning of the new millennium, biomedical research on microRNAs (also known as miRNA or simply miR-) has gained significant attention as cardinal elements in regulatory gene machinery. In terms of classification, miRNAs belong to small non-coding RNAs (about 22 nucleotides of a single-stranded molecule), generally well preserved between several organisms, involved in the regulation of gene expression by base pairing to mRNAs. According to the most recent miRNA database (miRBase v. 22.1, October 2018, http://mirbase.org), almost 2700 mature miRNAs have been annotated in the human species with some of them highly expressed in brain transcriptomes [1]. miRNA biogenesis takes place via RNA polymerase II or III in the shape of a primary transcript called pri-miRNA, that is further processed in the nuclear compartment into a pre-miRNA by the ribonuclease Drosha, with the cofactor binding protein DGCR8 Microprocessor Complex Subunit (DGCR8). The pre-miRNA recognition by the specific exportin 5 is responsible for the cytoplasmic translocation where the complex Dicer1, Ribonuclease III/TAR RNA binding protein (Dicer/TRBP) gives rise to a 21–22 nucleotide duplex that, eventually loaded via the Argonaute protein, is integrated as mature miRNA into the RNA induced silencing complex (RISC). miRNAs mostly impair target mRNAs or abolish their translation by binding to complementary sequences in the 3’ untranslated region (3’UTR) [2]. However, beyond their repressor activity, upregulation of specific mRNA targets upon 5’UTRs or coding regions has been ascribed to miRNAs [3,4,5,6].

Even a more focused search using the terms “miRNA and development” displays almost 24,000 papers suggesting a plethora of functions already orchestrated from very early steps of mammalian embryogenesis. Therefore, the main goal of this review is to address the role of miRNAs during human ontogenesis, with particular emphasis on the multiple pathways leading to the acquirement of neural stemness (both normal and tumoral) versus neuronal differentiation and subtype specification. Nevertheless, ethical technical constraints have so far limited studies during human brain development in vivo and therefore the dynamic of miRNA interactions has been mainly investigated in vitro by means of human embryonic stem cells (hESCs) and their variants in shape of neurospheres [7,8], conditionally immortalized human neural stem cell (hNSC) lines, human pluripotent stem cells (hPSCs) [9] and finally the in vitro recapitulation of the whole brain in shape of organoids [10]. Using such inclusion criteria within the last two decades, we have noticed that, despite the number of miRNAs expressed in the nervous system overcoming any other system, particularly for driving neurogenesis and the gliogenesis process [11,12], very few miRNAs have been extensively studied (i.e., miR-9, miR-124, miR-125), not only with respect to the expression levels by means of miRNA array but also the induced signaling cascade leading to the target entanglement.

### 1.2. Human Normal NSCs vs. Neural CSCs 

The brain cancer stem cell theory proposes that brain tumors harbor a subset of cells characterized by self-renewal, a high migration rate and unlimited growth capable of driving tumor development and progression, as well as being responsible for tumor aggressiveness, recurrence and resistance to conventional chemo- and radiation therapies [13,14,15]. These cells, namely neural cancer stem cells (CSCs), can be defined as transformed stem cells deriving from the conversion of normal NSCs toward tumor-forming stem cells, often as a result of the accumulation of genetic mutations. Neural CSCs exhibit properties that mirror those of NSCs, such as self-renewal, maintenance of an organ or tumor and differentiation into several cell lineages, albeit in a dysregulated fashion [16,17].

Gliomas are infiltrative tumors originated from glial cells and represent the most common and deadly primary malignant brain tumor [18]. More than half of gliomas are glioblastoma multiforme (GBM, World Health Organization grade IV astrocytoma), the most frequent and aggressive of the central nervous system (CNS) tumors. Similar to the normal brain, GBM is characterized by a marked cellular heterogeneity, that includes a neural CSC population of so-called glioma stem cells (GSCs). Relying on this, GBM has been a prototypic tumor for the study of the biology of neural CSCs [18,19,20,21,22].

Gene expression profiling studies have shown that GBM tumors, as well as patient-derived GSCs, can be classified in three transcriptionally-defined and clinically relevant subtypes: proneural (PN), mesenchymal (MES) and classical (CL), although individual GBM tumors are far from being uniform and actually maintain substantial cellular heterogeneity. Among these subpopulations, MES GSCs are more biologically aggressive and show higher levels of autophagic activity, along with heightened resistance to treatments than PN GSCs, while CL GSCs show a transitional hybrid profile [23]. GSCs are characterized by significantly increased expression levels of the neural stem cell lineage markers nestin, Prominin 1 (CD133), Nanog, SOX2, as well as Hematopoietic Cell E- and L-Selectin Ligand (CD44) and Octamer-Binding Transcription Factor 4 (OCT4), and demonstrate a marked ability to form neurospheres in particular conditions. The excessive heterogeneity is a main factor that accounts for chemotherapy resistance. GSCs are a dynamic population, maintained in discrete niches, with the highest concentration being at the infiltrating tumor edge in vivo [13]. Since these cells are responsible for the long-term maintenance of tumor growth, their close involvement in metastasis has been predicted [24,25].

**Figure 1:**
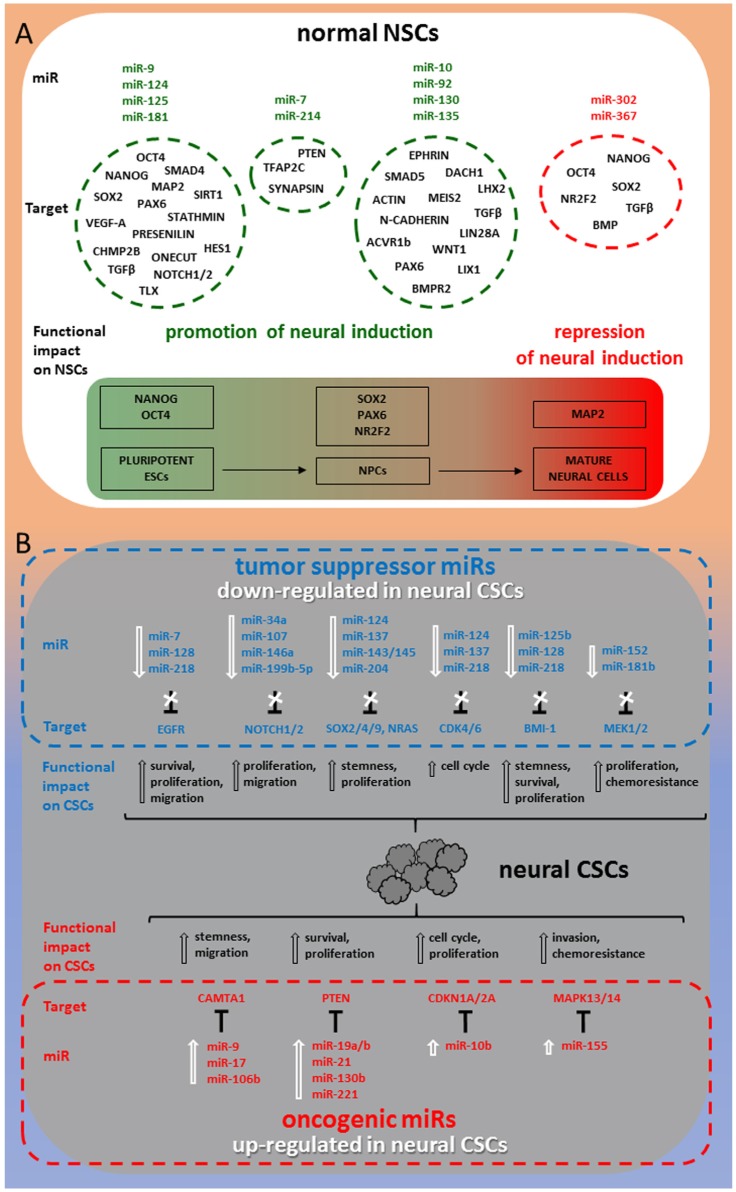
miRNA-target regulatory network in neural stem cells (NSCs). In normal NSCs, miRNAs are intercalated from embryonic stages along the differentiation pathway toward mature neural lineages (**A**). In neural cancer stem cells (CSCs), miRNAs operate as either tumor suppressors or oncomiRs governing the maintenance of CSCs and the homeostasis of neuronal/glial tumors (**B**).

Figure 1 shows a synoptic diagram highlighting the best characterized miRNAs in relation with their target molecules and dependent functional impact on both normal NSCs development and transition to CSCs in tumors of neuronal/glial origin.

## 2. miRNAs in Human Normal NSCs

### 2.1. miR-9, miR-124, miR-125, miR-181

The pioneer study by Delaloy and collaborators [26,27] has addressed the interest toward the neurobiology of miR-9 with a plethora of morphological, functional and genetic techniques, with the aim of following the early appearance, from 0 to 60 days in vitro (DIV), in human neural progenitor cells (hNPCs) derived from human embryonic stem cells. miR-9 was activated between 16–20 DIV during neurosphere formation and progressively increased until terminal differentiation as shown by in situ hybridization. Since the common precursor pre-miR-9-2 proportionally raised with the time course, both miR-9 and miR-9* were expressed accordingly, consistent with results obtained with miR-124, confirmed to be at high levels in postmitotic neurons [28]. By contrast, miR-367 was abundantly detected in hESCs and embryoid bodies (EBs) as a marker of pluripotent ESCs [29]. Meanwhile, the expression profile of cellular markers identified Nanog and OCT4 as the only represented proteins during the first week of differentiation, while Sex Determining Region Y-Box 2 (SOX2) and Paired Box 6 (PAX6) were dominant after the 2nd week. miR-9 was also expressed in Microtubule Associated Protein 2 (MAP2) positive postmitotic human neurons and, at lower levels, in human astrocytes. Remarkably, loss of miR-9 activity had a dramatic effect for the size reduction of neurospheres 5 days after its knockdown, but in the absence of hNPCs apoptosis in newly formed neurospheres, as assessed by cell death assay, leading to the conclusion that only cell proliferation was affected and endorsed by migratory inhibitory signals. In that context, the stability of progenitor features indicated that migratory ability is a distinct phenomenon, possibly from early differentiation. In vivo experiments following hNPCs transplantation at the injury site of the adult brain confirmed the described results. Moreover, qRT-PCR experiments showed a direct correlation of migratory behavior of early hNPCs with the mRNA upregulation for stathmin, Charged Multivesicular Body Protein 2B (CHMP2B), and Sirtuin 1 (SIRT1). Additional studies [30] offered new insights to understand the first stages of embryonic neurulation in vitro, extending, beyond miR-9, to miR-124 and the two isoforms of miR-125a and 125b the role of determinant players for neural lineage commitment triggered by the inhibition of Transforming Growth Factor β (TGFβ)-like molecule-mediated pathways. Such induction model has been exploited to validate the lack of miR-9 and miR-124 expression until day 8 of hESCs neural differentiation. Conversely, the activation of miR-125a and miR-125b was anticipated by a clear peak detected at 2 DIV of hESC commitment to the neural lineage. The more efficient induction of neural phenotype (over 80%) estimated by immunocytochemical detection of early neural markers PAX6 and SOX2 was achieved by the coadministration of SB431542 and noggin, which are both inhibitors of TGFβ-like molecules. On the contrary, the co-withdrawal of the same inhibitors determined the expression of the ESC markers, OCT4 and Nanog. Using the same induction paradigm, it was finally ascertained that the two isoforms miR-125a and miR-125b were only triggered by the concomitant activity of the above inhibitors, favoring the involvement of activin and the Bone Morphogenetic Protein (BMP) signaling pathway. Further transfection experiments using antago-miR-125 and pre-miR-125 complexes provided reliable proof of selective recruitment of miR-125 for neural lineage entry by blocking alternative phenotype options as previously published [31,32]. Moreover, further analysis of possible direct target of miR-125 were indicative of SMAD Family Member 4 (SMAD4) as a responsible element for PSC lineage achievement. Further studies carried out in the field of miR-125b [33] have disclosed an additional function that is shared with miR-181 that specifically fosters the selection of newly formed dopaminergic neurons. This therefore suggests that other brain specific miRNAs could be expressed in order to tag several neuronal subtypes by their neurotransmitter content.

With regard to miR-9, the Brustle group has put forth significant effort to elucidate the reciprocal interaction between this miRNA and Notch Receptor/Hes Family BHLH Transcription Factor (NOTCH/HES) [34]. Specifically, the bifunctional brain-enriched miR-9/9* have been explored in stably transduced human cell lines lt-NES (derived from H9.2 ES cell lines), starting from the statement that miR-9 complex is implicated in the transition from stemness proliferative state to neuronal maturation by means of a significant increase of the same miRNAs [35]. In order to demonstrate the hypothesis of whether NOTCH1, NOTCH2, and HES1 could be preferred targets of miR-9 and miR-9*, researchers overexpressed the genomic sequence of the miR-9_1 locus in lt-NES cells in a doxycycline-inducible manner and assessed quantitative variations in the expression of those proteins by western blot (WB) and real-time qRT-PCR analyses. After 4 days, results showed a robust increase in the expression of mature miR-9 and miR-9* in basal conditions for stemness maintenance in presence of Epidermal Growth Factor (EGF) and Fibroblast Growth Factor 2 (FGF-2). As a matter of fact, both β III-tubulin and nestin levels were almost stable in miR-9/9* overexpressing cultures as well as NOTCH1 quantity, while a significant decline was observed in NOTCH2 mRNA and NOTCH2 protein variants. Likewise, miR-9/9* overexpression was able to induce a dramatic downregulation of both HES1 transcript and protein. These results have been duplicated using small-molecule neural precursor cells (smNPCs) that grow as small neural rosettes reminiscent of the early neural tube [36], to confirm that they were independent from the chosen neural stem cell system. Therefore, smNPCs transduced with miR9-9* lentivirus already showed a comparable downregulation of NOTCH2 and HES1 mRNA after 48 hr of doxocycline-induced overexpression. Combining the described results, the same authors proposed an intriguing HES1-miR-9 oscillation-like model that makes possible proliferation and differentiation in alternate periods. Moreover, the luciferase method provided the ultimate confirmation that NOTCH2 and HES1 are direct targets of miR9-9* activity in hNSCs. In addition, previous data showing direct correlation between Presenilin 1 absence in mice and miR-9 downregulation [37,38] supported the paradigm that a ɣ-secretase inhibitor was responsible for downregulating miR-9 expression in lt-NES cells, meanwhile reducing the expression of the known NOTCH downstream target, Hes Related Family BHLH Transcription Factor With YRPW Motif 1 (HEY1). 

However, considering the clinical and therapeutic consequences, the most fascinating feature ascribed to miR-9 is the coupling function with regard to neurogenesis and angiogenesis in human brain developmental stages, by regulating neuronal Vascular Endothelial Growth Factor A (VEGF-A) expression [39]. This study used purified neural stem cells from 14 week-old human fetal brains and human cortical spheroids (HCSs, 3D cultures resembling the developing human cerebral cortex) generated from human induced Pluripotent Stem Cells (iPSCs), as previously described [40]. Taking into account the already exposed role of miR-9 that orchestrates stemness and differentiation homeostasis, and its association to cancer vascularization in vitro [41], a potential role for this miRNA in physiological angiogenesis in vivo was proposed, after identification of mir-9 expression by means of in situ hybridization in NSCs in the SOX2+ proliferative zone of the developing human nervous tissue (cortical spheroids). Here, the authors came to the conclusion that miR-9 might modulate angiogenesis in vivo by decreasing the stability of T Cell Leukemia Homeobox (TLX) and One Cut Homeobox (ONECUT) mRNAs to dampen VEGF-A signaling. The constitutive neuronal expression of both TLX and ONECUT using a neuronal driver gave rise to vasculature defects similar to neuronal VEGF-A expression, and is direct evidence of their ability to control VEGF-A transcription in neurons downstream of miR-9, thereby conveying new hope for stroke and brain tumor regenerative therapies [39].

### 2.2. miR-7, miR-214

As already shown in this review, miRNA array technology is a powerful tool for simultaneous identification of several induced or repressed miRNAs in the course of neuronal differentiation and terminal maturation. Additional research has highlighted the importance of the upregulating brain specific miR-7 and miR-9 with the concomitant miR-214 downregulation, as assessed by real time PCR [42] for the progression of hESCs into functional neurons. miR-214 is well known as one of the cell cycle-related miRNAs and many reports have documented its upregulation in tumor cells [43,44]. Although the molecular fine tuning of the above miRNAs did not change the expression of the most recognized markers of mature neurons, such as β III-tubulin expression or neurofilament formation, miR-7 overexpression was able to potentiate synapsin expression in the derived neurons and therefore positively influence the neurite outgrowth and synapse formation.

### 2.3. miR-302, miR-367

The components of the miR-302 family have drawn scientific attention because of their involvement in the control of cell proliferation and cell fate that, not remarkably, are again linked to the activation or repression of the transcription factors OCT4, Nanog and SOX2. In particular, it has been demonstrated that OCT4 and miR-302 cooperate for modulating their target gene Nuclear Receptor Subfamily 2 Group F Member 2 (NR2F2) [45] function in the differentiating hESC that can intensify the transcriptional activity of the full-length NR2F2. The reciprocal control is performed both in the undifferentiated state but also during the differentiation stages, according to a peculiar method: in the stemness condition, both OCT4 and the OCT4-induced miR-302 directly inhibit NR2F2 at the transcriptional and post-transcriptional level, respectively. By contrast, NR2F2 directly weakens OCT4 interaction during differentiation. Chromatin immunoprecipitation (ChIP) showed that OCT4 was also linked to the NR2F2 locus, downstream of exon 1 of the NR2F2-203 isoform. Taken together, these results showed that in pluripotent hESCs, the NR2F2 gene is transcriptionally repressed by OCT4, but it is subjected to a quick activation upon release of OCT4 from the promoter. Moreover, NR2F2 is also repressed at the post-transcriptional level by the OCT4-activated miR-302. Interestingly, NR2F2 expression accurately overlapped the pattern of PAX6 temporal activation, supporting the statement that miR-302 gene is directly triggered by OCT4 in ESCs [46]. A recent study [47] dealing with the miR-302 cluster, which encodes for miR-302a/b/c/d and miR-367, was able to identify a set of 146 high-confidence targets corresponding to a wide range of functional categories such as cell proliferation homeostasis, chromatin organization, vesicle transport, actin cytoskeleton and extracellular matrix constituents. These heterogeneous properties are under the control of the overall inhibition of neural differentiation (high content of miR-302) and affect trophectodermal fate, which besides the recognized regulation of TGFβ, puts into action BMP signaling.

### 2.4. miR-10, miR-92, miR-130, miR-135 

Jönsson and collaborators [48], by miRNA array analysis, examined human fetal samples encompassing both hNPCs and floor-plate cells of a forebrain (FB), midbrain (MB) and hindbrain (HB). They found that miR-10 was constitutively and massively expressed in the HB and spinal cord (SC) and they succeeded in isolating 89 high-confidence miR-10 target genes, enriched for functions in transcription, actin cytoskeleton and ephrin receptor signaling. miR-10 was suggested as the key regulator in the caudalization process of hNPCs of different subtypes (see also miR-125b and mir-181 for a role in establishing different human neuronal subtypes). In addition, miR-92b-3p and miR-130b-5p were identified as two miRNAs highly expressed in FB, MB and HB cells compared with hESCs. Finally, two members of the miR-10 family, with dual function in brain and cancer development [49], were found abundantly and exclusively in HB cells. The analysis revealed that miR-92 family segregates into FB, MB neuroepithelium (NE) and MB floor-plate cells, giving a significant quantitative contribution to all miRNA reads. Moreover, miR-10 family members have a unique spatial regulation, resulting in a very high-level expression only in the HB. Therefore, on the basis of miRNA-seq data, it is feasible to suggest that different developing human brain regions can be classified according to their miRNA-expressing profile. Data from the miRNA array of developing human brain samples displayed robust quantities of miR-10a and miR-10b in the posterior regions, particularly the spinal cord, at all the developmental time points analyzed, but it was not detectable in any FB or MB samples, confirming a role for miR-10 in caudalization of hNPCs. Finally, miR-135b was found to be recruited during neuroectodermal development and that ectopic expression of miR-135b in hESC promoted differentiation toward NE. miR-135b switched neural conversion by targeting those elements of the TGFβ and BMP signaling pathways. Moreover, PAX6 was responsible for activating several transcription factors implicated in neural development as well as miR-135b in hESC lines H1 and H9. In particular, miR-135b was activated as early as day 2 and its expression faithfully mirrored the activation profile of PAX6 during the time course of differentiation and irrespective of the ESC line or neural induction method used. Transfection of hESC with miR-135b reproduced significantly induced neural genes such as PAX6, LIM Homeobox 2 (LHX2), Limb and CNS Expressed 1 (LIX1), Dachshund Family Transcription Factor 1 (DACH1), Meis Homeobox 2 (MEIS2) and N-CADHERIN as compared to the control transfection. Although OCT4 levels had no variation tendency, miR-135b overexpression downregulated the pluripotency marker Nanog, indicating that miR-135b can be a negative modulator of pluripotency signals by targeting Bone Morphogenetic Protein Receptor Type 2 (BMPR2), TGFβ, SMAD5, and Activin A Receptor Type 1B (ACVR1b) by directly binding to recognition sites in their 3’UTR. Despite transcript levels of all four candidates not showing significant changes, the suppression of BMPR2, SMAD5 and ACVR1b at the protein level was directly linked to the presence of miR-135b [50].

Very recently, some impressive works have been reported that explore the miRNA profiling unraveling the regulators of the neural differentiation of human iPSCs. Specifically, during the transition, miR-10, miR-30 and miR-9 families are involved in the upregulation, while miR-302 and miR 515 families are subjected to downregulation [51], including an evolutionary young miRNA, miR-1290, found to be crucial for neuronal differentiation in normal and autism spectrum disorder (ASD) affected brain [52].

## 3. miRNAs in Human Neural CSCs

Extensive efforts have been made to elucidate the genetic circuits that govern the malignant transition of NSCs toward highly invasive GSCs, in terms of mRNA microarray in glioma tissues. However, since mRNA expression largely reflects the consequences of transcriptional regulation, recent studies are aimed at elucidating the programs, particularly those controlled by miRNAs, that guide the NSC malignant transformation and the key pathways involved in this transition [13].

The maintenance of GSCs is largely orchestrated by epigenetic changes, such as those induced by miRNAs, that affect several aspects of GSCs biology, either by regulating gene expression or by post-transcriptionally reducing mRNA stability and suppressing mRNA translation [53,54,55]. In the past decade, the analysis of miRNA expression profiles using microarray technology has unraveled broad differences in miRNA signatures of human GSCs compared to NSCs, and the dysregulation of miRNA expression has been closely associated with the origin and progression of cancer [13]. Each miRNA can be involved in several signaling pathways regulating GSC biology by restraining the mRNA of different target genes.

Under different cellular contexts, miRNAs may have distinct biological behaviors functioning as either tumor suppressors or oncogenes (oncomiRs), by suppressing oncogenic mRNAs or tumor suppressive mRNAs, respectively. In addition, miRNAs can modulate tumor-modifying extrinsic factors, such as cancer-immune system interactions, stromal cell interactions, oncoviruses, and sensitivity to therapy. Ultimately, the balance between all of these processes determines if a specific miRNA produces a net tumor suppressive or oncogenic effect [54].

The balance between neural CSC self-renewal and differentiation is often driven by niche components including adhesion molecules, such as Junctional Adhesion Molecule A (JAM-A), involved in CSC-niche interactions that sustain CSC maintenance. Several studies have linked neural CSC-niche signaling to the miRNA regulatory network, that is altered in GBM and can be targeted to attenuate GSC self-renewal.

Here we review the best studied miRNAs, dysregulated specifically in neural CSCs compared with non-stem brain tumor cells and NSCs, highlighting the impact on their putative target genes and the ultimate effect on neural CSCs fate. It is also our aim that the reader gains insight into the power of next generation miRNA-targeted therapies against human brain tumors.

### 3.1. Tumor Suppressor miRNAs in Neural CSCs

Significantly downregulated expression of miRNAs, referred to as tumor suppressor miRNAs, has been found in glioma, medulloblastoma (MB), meningioma tissues and cell lines, and notably in patient-derived neural CSCs. The literature investigation reveals how their decreased expression, specifically in neural CSCs, has been associated with enhanced tumor growth, invasion and poor patient outcome. Conversely, experimental transfection of these tumor suppressor miRNAs into neural CSCs has been shown to inhibit the viability and proliferation of CD133+ CSCs, significantly impairing tumorigenicity and invasion.

Among the tumor suppressor miRNAs, miR-7 is significantly diminished in GSCs, promoting the proliferation and migration of this cell population. Instead, its ectopic expression increases cell death by inhibiting several signaling pathways downstream of Epidermal Growth Factor Receptor (EGFR) [56,57,58].

miR-23b expression is markedly reduced in GSCs, as compared with matching non-stem U87 GBM cells. However, restoration of miR-23b expression in GSCs induces cell cycle arrest and inhibition of cell proliferation by downregulation of its target High Mobility Group AT-Hook 2 (HMGA2) [59,60].

miR-34a is downregulated in GSCs and in MB CSCs, resulting in increased cell survival and proliferation, invasion, and strongly reduced CSC differentiation. Conversely, miR-34a experimental induction exerts tumor suppressive effects in CSCs by inhibiting BCL2 Apoptosis Regulator (BCL2) and NOTCH1/NOTCH2 genes and boosting DNA damage responses [57,58,59,60,61]. The NOTCH1 pathway is also a direct target of the tumor suppressor miR-146a which, upon re-expression, can inhibit GBM development by reducing GSCs migration [59].

miR-107 functions as a tumor suppressor by reducing the expression of NOTCH2, CD133, nestin and Matrix Metallopeptidase 12 (MMP-12) in GSCs, impairing stem cell proliferation and invasion. Its downregulation in GBM is also strongly associated with poor patient outcome [59,62].

miR-124 is a unique neural miRNA downregulated in GSCs, resulting in prompted cell stemness. Transfection of miR-124 instead induces differentiation of CD133+ cells by targeting the Polypyrimidine Tract Binding Protein 1 (PTBP1), SOX9 and RE1-Silencing Transcription Factor (REST) pathways, and leads to G1 cell cycle arrest in glioma and MB cells, by inhibiting its targets Cyclin-Dependent Kinase 4 (CDK4) and CDK6, confirming that miRNAs play a pivotal role in the regulation of the biology of CD133+ cells from gliomas [58,59,63,64]. Moreover, miR-124 attenuates neurosphere formation and stem cell markers expression by targeting NRAS Proto-Oncogene, GTPase (NRAS), Pim-3 Proto-Oncogene, Serine/Threonine Kinase (PIM3) and Snail Family Transcriptional Repressor 2 (SNAI2) [56].

miR-125b and miR-128, two of the major miRNAs described to be downregulated in GBM, show a remarkably lower expression in CD133+ GSCs, as compared with CD133-cell populations. Upon expression, they both inhibit GBM growth by decreasing the self-renewal and proliferation of GSCs, via BMI1 Proto-Oncogene, Polycomb Ring Finger (BMI-1) downregulation, thereby exerting a pro-apoptotic role [65,66,67,68,69,70,71]. Moreover, miR-128 represses GSCs growth and mediates their differentiation by targeting the oncogenic EGFR/Platelet-Derived Growth Factor Receptor/AKT Serine/Threonine Kinase (EGFR/PDGFR/AKT) signaling [68,72,73]. Rooj et al. have recently shown that the expression of miR-128, in parallel with the signature of its dependent target genes, can stratify the PN from the MES GSC subtype, with the lowest miR-128 levels in MES GSCs, the highest in PN GSCs, and a transitional signature in CL GSCs. Subtype-specific gene signatures also seem to separate patients into two clusters, MES and PN/CL-like, which are significantly predictive of the disease outcome [74].

miR-134 is downregulated in glioblastoma, oligodendroglioma tissues and in glioblastoma cell lines compared with normal brain tissues. A confirmed miR-134 target is Nanog, with miR-134 restraining both the mRNA and protein expression of this transcription factor, thereby preventing cell proliferation, migration, and inducing apoptosis. The loss of miR-134 is therefore involved in brain tumorigenesis and progression [75,76].

miR-137 expression is significantly reduced in GSCs compared with NSCs, while its induced overexpression promotes neuronal differentiation of both cell types. Gene ontology analysis has identified cell cycle as the main process affected by miR-137, with oncogenes such as KIT Proto-Oncogene, Receptor Tyrosine Kinase (c-KIT), AKT2, TGFB2, CD24 Antigen (Small Cell Lung Carcinoma Cluster 4 Antigen, CD24), Cell Division Cycle 42 (CDC42), CDK6, and Y-Box Binding Protein 1 (YBX1) being negatively regulated [77]. miR-137 significantly decreases the self-renewal of GSCs and the stem cell markers OCT4, Nanog, SOX2, and Sonic Hedgehog Signaling Molecule (SHH). In addition, transfection of cells with miR-137 decreases the expression of RTVP-1 Related to Testis-Specific, Vespid, and Pathogenesis Proteins 1 (RTVP-1), a novel target of miR-137 regulating GSC stemness [64,78]. Since miR-137 shares a large number of target genes with similar miRNAs, such as miR-7, -124 and -128, it is likely that they may act in a coordinated manner to boost their regulatory effect during gliomagenesis [77].

Significantly decreased levels of the miR-143/145 cluster in GSCs have been correlated with shorter median patient survival rates and high levels of ATP-Binding Cassette Subfamily G Member 2 (ABCG2), suggesting that the miR-143/145 cluster is involved in the regulation of GSC stemness properties [56,79,80]. miR-143 inhibits proliferation of GSCs under hypoxic conditions and decreases their tumor formation capacity in vivo [59]. miR-145 has JAM-A as a direct target, but its signaling system extends to pluripotency factors such as SOX2, OCT4, Nanog, which are downregulated upon miR-145 transfection into GSCs [22,81].

miR-152 expression levels are significantly reduced in glioma tissues with different grades and in sphere-forming GSCs, as compared with normal brain tissues. A direct and functional target of miR-152 is the transcription factor Krüppel-like Factor 4 (KLF4), which is implicated in the establishment, maintenance of pluripotency and in controlling essential cellular processes such as proliferation, differentiation, and migration. The reduction of miR-152 expression is therefore critically involved in GSC biological behavior and glioma development, fostering tumor growth. Restoring miR-152 expression, by downregulating KLF4, inhibits the expression of Galectin 3 (LGALS3) and indirectly attenuates the activation of Mitogen-Activated Protein Kinase Kinase 1/2 (MEK1/2) and Phosphoinositide-3-Kinase C (PI3K) signaling pathways, resulting in reduced cell proliferation, migration and invasion, as well as increased apoptosis [82].

miR-153 expression is downregulated in GBM tissues relative to normal brain tissues and in CD133+ cells relative to CD133- cells. However, the experimental gain of miR-153 inhibits GSCs growth and stemness properties by impairing self-renewal ability and inducing differentiation and apoptosis [13,59].

miR-181b results reduced by more than 100-fold in GSCs when compared to GBM tumor cells, which correlates with shorter median patient survival times. On the contrary, induced upregulation of this miRNA suppresses cell proliferation and induces sensitivity to the chemotherapeutic drug temozolomide (TMZ) by directly binding to MEK1, which is involved in the Mitogen-Activated Protein Kinase (MAPK) pathway [83].

miR-199b-5p expression is lost in MB patients, as a result of epigenetic silencing occurring during carcinogenesis, and is associated with a metastatic tumor phenotype. Re-expression of miR-199b-5p represses HES1 and different genes of the NOTCH signaling pathway, thereby impairing the self-renewal capacity of CD133+ CSCs [66].

miR-203 is expressed at lower levels in CD133+ GSCs compared with CD133- cells and normal brain tissue. Upon its transfection, it acts as a stemness-inhibiting miRNA leading to increased expression of Glial Fibrillary Acidic Protein (GFAP) and MAP2, indicating a tendency towards GSC differentiation [84].

miR-204 is markedly downregulated in both GSCs and NSCs. Restoring its expression suppresses GSC self-renewal and migration by targeting the stemness-governing transcriptional factor SOX4 and the migration-promoting receptor EPH receptor B2 (EphB2), leading to increased overall patient survival [59].

miR-211, downregulated in glioma, when experimentally overexpressed, leads to the activation of the intrinsic mitochondrial/Caspase-9/3-mediated apoptotic pathway in GSCs [59].

miR-218 is significantly downregulated in glioma as well as in MB and its low expression correlates with tumor aggressiveness. Once miR-218 expression is restored, it decreases the expression of its functional downstream target BMI-1 oncogene and blocks the self-renewal of GSCs. In MB, miR-218 transfection restores its tumor suppressor properties primarily through suppression of its target CDK6 [59,85].

miR-608 is one of the newly discovered miRNAs significantly downregulated in GSCs, linked to an increase of the Macrophage Migration Inhibitory Factor (MIF) gene and protein. On the contrary, miR-608 overexpression negatively regulates the expression of MIF, attenuating proliferation, migration, invasion, and inducing apoptosis of GSCs [86].

Downregulation of miRNAs in glioma may be controlled by diverse epigenetic mechanisms, including DNA methylation, histone modification, or post-transcriptional processes. Of these, promoter DNA methylation is related to the silencing of miRNAs possessing promoter-associated CpG islands. Recently, increasing studies have described the role of DNA methylation of tumor suppressor miRNAs, including miR-211, miR-204, miR-23b, miR-145 and miR-137 in GSCs [59].

In addition, RNA-binding proteins (RBPs) can modulate miRNA functions. This can be seen in the case of the IMP2 protein, which binds to a subset of target transcripts, including Cyclin D1 (CCND1), Paternally Expressed 10 (PEG10), HMGA1, HMGA2 and Insulin-like Growth Factor 2 mRNA-Binding Protein (IMP3), and protects them from silencing by the let-7 miRNA family members. let-7 miRNAs play a central role in promoting cell cycle arrest and differentiation by silencing stemness genes. Nevertheless, despite their high expression in GSCs, they are not able to exert their tumor suppressive function due to the protective role of IMP2 that sustains tumorigenicity and stemness in GSCs [87].

### 3.2. OncomiRs in Neural CSCs

Conversely, upregulated expression of miRNAs, identified as oncogenic miRNAs, results in enhanced cell invasion, self-renewal and dramatic reduction of apoptosis and differentiation of neural CSCs.

By the linear models for microarray data (LIMMA) approach, Sana et al. recently revealed a high number of miRNAs upregulated in GSCs compared with paired non-stem GBM cell cultures, which positively correlated with SOX2 and nestin expression, suggesting their close association with the stem-cell-like phenotype of GSCs. It is noteworthy that, among the differentially expressed miRNAs, a seven-miRNA signature (miR-9-3p, -93-5p, -106b-5p, -153-3p, -301a-3p, -345-5p, and -652-3p) was associated with a significantly lower overall survival of patients [88].

miR-9/9*, -17, and -106b are abundant in CD133+ GSCs and induce stem cell generation, migration and invasiveness, properties which can also be found in NSCs. Inhibition of these miRNAs results in reduced neurosphere formation and induction of cell differentiation by direct targeting of Calmodulin-Binding Transcription Activator 1 (CAMTA1). Interestingly, Inhibitor of Differentiation 4 (ID4) induces de-differentiation of human glioma cells towards a GSC phenotype and enhances SOX2 expression by suppressing miR-9* [56,59,61,89].

miR-17-5p, -19a-3p, -19b-3p, -21-5p, -130b-3p, -221-3p, and -222-3p have been found overexpressed in GSCs and correlated with reduced expression of their common target Phosphatase and Tensin Homolog (PTEN), leading to anti-apoptotic effects, promoting tumor growth, and sustaining a stem-cell-like phenotype [89,90]. miR-221/222 directly target Cyclin Dependent Kinase Inhibitor 1/2 (KIP1/2) to reduce apoptosis and regulate cell cycle progression [58].

miR-10b is a unique oncomiR highly expressed in GSCs, but absent in NSCs and in normal brain astrocytes, which is involved in the dissemination of GBM. miR-10b regulates cell cycle by targeting Bcl-2-like Protein 11 (BCL2L11/Bim), Transcription Factor AP-2 Gamma (TFAP2C/AP-2γ), Cyclin Dependent Kinase Inhibitor 1A (CDKN1A/p21) and CDKN2A/p16, which normally protect cells from uncontrolled growth. In addition, miR-10b affects several mRNA splicing factors including Muscleblind-like Splicing Regulator 1-3 (MBNL1-3), Spliceosome Associated Factor 3, U4/U6 Recycling Protein (SART3) and Arginine and Serine Rich Coiled-Coil 1 (RSRC1), often by the non-canonical binding to their 5’UTRs. Inversely, miR-10b inhibition has been demonstrated to strongly impair GSC viability and proliferation [72,91,92,93].

Recent evidence indicates that miR-21 is a powerful oncomiR overexpressed in GSCs and is associated with unfavorable outcomes in GBM patients [62]. miR-21 acts as an antiapoptotic factor by targeting p53 and TGFβ [72]. Moreover, by inhibiting an entire network of oncosuppressor genes such as PTEN, Programmed Cell Death 4 (PDCD4), Reversion Inducing Cysteine Rich Protein with Kazal Motifs (RECK), Tropomyosin 1 (TPM1), it plays a pivotal role in promoting cell proliferation and invasion, and confers GSCs a chemoresistant phenotype [55]. Conversely, the miR-21 blockade by anti-miR oligonucleotides strongly disrupts tumor growth and enhances GSCs apoptosis, furthermore sensitizing GSCs to TMZ treatment [64,94]. Markedly, the upregulation of miR-21, corresponding to increased mRNA levels of the stem cell marker nestin, have also been found in atypical and malignant meningioma, in comparison to benign meningioma [95].

miR-93 is upregulated in both GBM cells and GSCs, although its oncogenic function appears controversial and, at least in part, dependent on the GSC subtype. The MES GSC subpopulation, characterized by pronounced basal autophagic activity sustaining tumor growth and survival, shows significantly lower levels of miR-93 compared with PN GSCs. However, miR-93 overexpression in this subpopulation leads to decreased expression of the MES GSC stemness markers Aldehyde Dehydrogenase 1 Family Member A3 (ALDH1A3) and CD44, increased expression of the differentiation marker Tubulin Beta 3 Class III (TUBB3), along with downregulation of autophagy regulatory genes, which results in attenuated cell growth and sphere-forming ability, as well as sensitization of MES GSCs to TMZ and radiation therapy [23].

miR-155 is an oncomiR expressed in GSCs nine times more than in NSCs and has been associated with poor overall patient survival. Its tumor promoting function mainly consists of promoting cell proliferation, metastasis and chemoresistance by suppressing Caudal-Type Homeobox 1 Protein (CDX1) and MAPK13 and MAPK14, respectively. Knockdown of miR-155 inhibits cell growth and invasion and sensitizes GBM cells to apoptosis induced by TMZ chemotherapy. Bioinformation analysis shows that one of the predicted targets for miRNA-155-5p is BMP, which regulates proliferation and differentiation of GSCs. [13,55,90,96].

Expression of miR-196a and -196b is very high compared with other overexpressed miRNAs in GBM and is associated with shorter overall patient survival [72]. Interestingly, miR-196a-5p acts as an oncomiR in GSCs, inhibiting the transcription factor Forkhead Box O1 (FOXO1) as its main target. FOXO1 binds and transcriptionally activates Phosphotyrosine Interaction Domain Containing 1 (PID1) and Migration and Invasion Inhibitory Protein (MIIP), that induce apoptosis and inhibit glioma growth and invasion [97].

miR-210 and miR-373 are upregulated in response to hypoxia conditions and play a vital role in GSCs’ adaption to hypoxia and survival. Interestingly, miR-210 promotes cell proliferation by targeting several genes such as MAX Network Transcriptional Repressor (MNT), Iron–Sulfur Cluster Scaffold Protein (ISCU) and Ephrin-A3 (EFNA3). Knockdown of miR-210 by a specific anti-sense sequence strongly inhibits stemness, proliferation, invasion, radioresistance and induces apoptosis, differentiation and cell cycle arrest in hypoxic GSCs, by partially rescuing the expression of MNT [98].

miR-455-3p is upregulated in GSCs and seems to have a silencing effect on SMAD2, that drives cell proliferation. Higher miR-455-3p expression levels are indeed associated with shorter median patient survival times and therapeutic resistance [83].

miR-1275 overexpression suppresses the expression of Claudin11, an important protein during oligodendroglial lineage differentiation, and sustains tumor cell growth. Inhibition of miR-1275 in GSCs, with the specific antisense oligonucleotide anti-miR-1275, has been demonstrated to increase the expression of Claudin11, in parallel with significant suppression of tumor growth [59].

### 3.3. miRNAs in GSC Exosomes

Pediatric high-grade gliomas (HGGs) are one of the most significant causes of morbidity and mortality among children, due to their aggressive clinical behavior, probably driven by their GSC component. GSCs, as well as normal NSCs, may release extracellular vesicles, called exosomes, carrying small non-coding RNAs, such as miRNAs, namely exosomal miRNAs. Exosomal transfer of miRNAs has been recognized as an important system of cell–cell communication for the exchange of epigenetic information, although little is known about the types of exosomal miRNAs released by GSCs and their role in HGG biology. Despite a few differentially expressed cellular miRNAs in normal fetal NSCs and GSCs, a significantly different repertoire of miRNAs has been observed in the exosomes released by pediatric GSCs compared with normal NSC-secreted exosomes. Notably, exosomial miRNA signatures can be unique in cancer patients. In particular, exosomal miR-1246 and miR-1290 have been demonstrated to affect expression of target genes in receiving cell lines, particularly downregulating the expression of glioma-associated tumor suppressors PTEN and Tet Methylcytosine Dioxygenase 3 (TET3) and up-regulating cancer-related genes such as SERTA Domain Containing 1 (SERTAD1) and SEC61 Translocon Gamma Subunit (SEC61G) [14]. These observations thus suggest that exosomes could play a role in the tumor microenvironment by means of targeting genes with a role in cell fate and tumorigenesis and finally influencing the tumorigenic properties of neighboring cells [14]. Moreover, the repertoire of exosomal miRNAs, along with the cellular miRNA profile of neural CSCs, might account for the tumor heterogeneity.

### 3.4. miRNAs in Human Melanoma

Melanoma is a tumor that develops by the malignant transformation of neural crest-derived melanocytes, typically in the skin but also in rare circumstances, in the mouth, intestinal tract, or in the uvea of the eye. Due to its frequency and aggressiveness, it represents the most thoroughly studied neural crest-derived cancer [99]. In the bulge area of hair follicles in the dermis, a reservoir of melanocyte stem cells (MSCs) has been localized, which if mutated can proliferate and migrate abnormally, resulting in the development of melanoma [100].

Solid evidence reveals a differential miRNA expression in melanoma cell lines and lymph node metastases in comparison to normal melanocytes, with under-expression of miR-192, let-7i, -194, -211, -602, -582, -454-3p, -132, -509, and over-expression of miR-126 and miR-801.

Distinct miRNA expression profiles between primary or metastatic melanomas and benign nevi were also reported, with miR-203 as one of the top miRNAs under expressed in tumor samples, but upregulated in serum and in melanospheres, as a melanoma stem cell model. In metastatic melanoma cell lines, miR-203 enhances proliferation, stemness potential, number and size of melanospheres, tumorigenicity, by upregulation of SOX2, KLF4 and OCT4, as the main transcription factors sustaining pluripotency. Moreover, miR-203 induces cell motility by upregulating genes involved in epithelial-to-mesenchymal transition (EMT). The dysregulation of miR-203 levels may thus play an important role in the induction of melanoma stem cells and in preparing cancer cells for metastasis [101,102]. On the contrary, in GSCs, miR-203 demonstrates the potential to reduce stemness, confirming the dual oncogenic/suppressive role of miRNAs in different tumors [84].

Consistent with the cell heterogeneity observed in tumors, specific miRNA signatures have been shown in melanoma cells grown as monolayers, as compared with melanoma cells forming spheroids. Spheroids contain a sub-population of cancer cells exhibiting stem-like properties: increased potential for self-renewal, high ability to differentiate along the mesenchymal lineage, and enhanced expression of human embryonic stem cell pluripotency markers, such as SOX2, Nanog and OCT4. Eight miRNAs (miR-1301, -182-5p, -191-5p, -1915-3p, -378d, -3934, -4767, -542-3p) are upregulated in spheroid cells which, by mRNA-target prediction models, show that they are directly involved in regulating the expression of several genes belonging to the Wingless-Related Integration Site (WNT) signaling pathway. In particular, miR-542-3p has also been described as a tumor suppressor in neuroblastoma, since it reduces tumor growth and invasive potential [103].

miR-455 has been identified as downregulated in primary and metastatic melanomas, in comparison to benign nevi. Since one of its putative target genes, PAX6, is crucial for self-renewal and differentiation of NSCs, the loss of miR-455 and its subsequent effect on PAX6 expression may disrupt the normal progression of melanogenesis, resulting in an immature melanocyte phenotype with increased migratory capacity and enhanced metastatic potential [104].

Research has shown that, in WM-115, NA8, SK-MEL, Me67, A375, D10 human melanoma cell lines, oncomiR-10b, -21, -520c, -373 and the tumor-suppressor miR-200c, dysregulated in sphere-forming melanoma stem cells, are the most important miRNAs in the modulation of EMT, migration, invasion, and consequent tumor progression [105,106].

Deregulation of miRNAs expression in MSCs has been shown to be involved in the metastatic process and to be associated with patient survival and mutational status. Low expression of miR-191, as well as high expression of miR-193b, let-7i and -365 has been associated with poor survival [105,107,108]. Moreover, high expression of miR-193b has been correlated with a high risk of metastasis in uveal melanoma [105].

### 3.5. miRNAs as Diagnostic, Prognostic and Predictive Tools

To this end, miRNA signatures have been correlated with tumor stage, progression, and prognosis of cancer patients. Specifically, the differential expression of single miRNAs, including miR-203, -210, -125, has been used to distinguish GBM from low-grade gliomas and healthy controls and has been related to clinical outcome [88,105,107,108,109,110]. The increasing speculation on miRNA expression profiling and the relationship with cognate target genes will provide new clues for the mechanisms involved in the pathogenesis of neuronal and glial tumors as well as diagnostic and prognostic tools [13,89], along with being predictive markers of the response to chemotherapy. Moreover, the miRNome analysis of brain CSCs compared to normal NSCs would aid in identifying the normal brain cells prone to tumor growth. Moreover, molecularly based models of risk assessment would improve the standard staging criteria incorporating miRNA expression profiles into such models. Identification and subsequent targeting of mechanisms responsible for the maintenance of CSCs, in combination with current GBM treatments, may have a synergistic therapeutic effect and could therefore improve patient prognosis.

Pediatric brain tumors differ vastly from adult tumors mostly in their epigenetic regulation, which appears to be more evident than in the adult counterparts. If a child develops a brain tumor before the age of 2 years, it is likely a result of genetic/epigenetic alterations that have induced tumorigenic transformation in NSCs within the developing brain, rather than due to a long-term carcinogen exposure that may be mutagenic in oncogenes or tumor suppressor genes [21,111,112]. For this reason, further studies would shed light on the epigenetic alterations that may be the driving force of pediatric brain CSC propagation and maintenance.

### 3.6. miRNAs and Their Therapeutic Implication

Current treatments for brain tumors target the bulk of cancer cells, but do not adequately target self-renewing CSCs [21]. To fulfill the eventual goal of developing CSC-targeted therapies, the identification of CSC-specific regulatory circuitries is required.

Nearly all GBM tumors recur following surgical resection and even treatment of patients with the routinely-used cytotoxic TMZ and radiation therapies. Recurrent tumors are enriched with GSCs, and there are no treatments that have shown consistency in limiting the growth of recurrent GBM. Relying on this, therapies aimed to antagonize the function of miRNAs that sustain GSC stemness/viability could represent an alternative and effective strategy to treat primary as well as recurrent GBM [23,113]. Resistance to therapy and tumor relapse are believed to reside in the high intratumoral heterogeneity and, in particular, in the highly plastic subpopulation of GSCs. GSC plasticity is a product of epigenetic and post-transcriptional regulation of gene expression, mostly by miRNAs, that determines cell hierarchies during neoplastic growth. Therefore, development of efficient miRNA targeting strategies is critically important. Studies focused on GBM subtype-specific miRNA and gene signatures will be crucial to better understanding the intratumoral heterogeneity and subpopulation dynamics relevant to tumor response to therapy and patient outcome, and to establishing effective personalized therapeutic treatments against GBM.

Gaining a better understanding of both the epigenetic and microenvironmental signals, responsible for brain tumor heterogeneity and plasticity, will offer a wider array of avenues for the development of therapies identifying and targeting CSCs within different tumor subtypes [21,114]. In particular, for the reasons explained above, pediatric brain tumors may benefit more from genetic and epigenetic targeted therapies [21].

Since a single miRNA can regulate the expression of multiple genes involved in diverse functions, and cancer is actually a disease with multiple gene aberrations, developing novel approaches to govern miRNA pathways uncovers exceptional opportunities for brain tumor treatment [115].

There is a growing interest in the role of miRNAs in the treatment of neuronal and glial tumors: the modulation of miRNA expression levels, particularly the inhibition of oncomiRs, holds great promise for supplying more effective therapeutic approaches to these malignancies [13,53,116]. Interestingly, the inhibition of oncomiRs, such as miR-10b, overexpressed in GSCs but lacking in NSCs, could compromise proliferation and survival of GSCs without affecting normal neural cells, thus providing a unique opportunity for specific and non-toxic therapy [93].

Several strategies have been developed in recent years to inhibit oncomiRs. Among them, a direct approach targets mature oncomiRs with an antisense sequence known as anti-miR (antagomiR), which could be an oligonucleotide or a miRNA sponge. In contrast, an indirect approach is to block the biogenesis of miRNAs by genome editing using the Clustered Regularly Interspaced Short Palindromic Repeats/CRISPR-Associated Protein 9 (CRISPR/Cas9) system or a small molecule inhibitor [55]. Recent studies have shown that silencing miR-21, overexpressed in GBM and responsible for promoting tumorigenesis, significantly enhanced the antitumoral action of the tyrosine kinase inhibitor sunitinib in GBM cellular models [18]. LNA-anti-miR-21 was shown to increase the treatment efficacy of a secreted variant of the cytotoxic agent Secretable form of Tumor Necrosis Factor-Related Apoptosis Inducing Ligand (S-TRAIL) on glioma. LNA-anti-miR-21 enhanced S-TRAIL-induced caspase activation and thereby the apoptotic response, and the combination led to complete eradication of gliomas in murine brain xenografted with human glioma U87 cells [117].

Nevertheless, the successful in vivo delivery of anti-miR oligonucleotides to brain tumors appears considerably challenging, since it will require the development of carriers that are not only able to increase bioavailability, but that can also overcome the blood–brain barrier and enhance target cell uptake, while sparing the normal tissues [115,118,119].

On the other hand, since the miRNA-mediated inhibition of pathways prompting stemness and oncogenic factors in GSCs would prevent tumor proliferation and invasion, the restoration of tumor suppressor miRNAs expression, leading to a balance towards cell differentiation, may represent an effective therapeutic approach in multi-modal therapy for poorly differentiated GBM. Since the expression and function of different tumor suppressor miRNAs, epigenetically inactivated in GSCs by aberrant DNA methylation [120], can be reversed by DNA hypomethylation treatment, this may suggest a further promising application in the treatment of CNS tumors.

Newly, the rationale of combinatorial miRNA strategies in anticancer treatments has been emerging. Among the most profoundly downregulated miRNAs in GBM, Bhaskaran et al. [121] identified a module of miRNAs, comprised of miR-124, -128 and -137, showing a pattern of clustered expression during neuronal differentiation, but simultaneously lost along gliomagenesis. The induced synchronized expression of the three miRNAs in this gene therapy approach, combined with chemotherapy, displayed significant anticancer synergism and resulted in a 5-fold increase in survival in murine GBM models, supporting the feasibility and promising effectiveness of the clustered approach in antitumoral therapies.

## Abbreviations

*ABCG2*	ATP-Binding Cassette Subfamily G Member 2
*ACVR1b*	Activin A Receptor Type 1B
*AKT*	AKT Serine/Threonine Kinase
*ALDH1A3*	Aldehyde Dehydrogenase 1 Family Member A3
*BCL2*	BCL2 Apoptosis Regulator
*BCL2L11/Bim*	Bcl-2-Like Protein 11
*BMI-1*	BMI1 Proto-Oncogene, Polycomb Ring Finger
*BMP*	Bone Morphogenetic Protein
*BMPR2*	Bone Morphogenetic Protein Receptor Type 2
*CAMTA1*	Calmodulin-Binding Transcription Activator 1
*CCND1*	Cyclin D1
*CD133*	Prominin 1
*CD24*	Small Cell Lung Carcinoma Cluster 4 Antigen
*CD44*	Hematopoietic Cell E- and L-Selectin Ligand
*CDC42*	Cell Division Cycle 42
*CDK*	Cyclin-Dependent Kinase
*CDKN1A/p21*	Cyclin Dependent Kinase Inhibitor 1A
*CDKN2A/p16*	Cyclin Dependent Kinase Inhibitor 2A
*CDX1*	Caudal-Type Homeobox 1 Protein
CHiP	Chromatin immunoprecipitation
*CHMP2B*	Charged Multivesicular Body Protein 2B
*C-KIT*	KIT Proto-Oncogene, Receptor Tyrosine Kinase
CNS	central nervous system
*CRISPR/Cas9*	Clustered Regularly Interspaced Short Palindromic Repeats/CRISPR-Associated Protein 9
CSC	cancer stem cell
*DACH1*	Dachshund Family Transcription Factor 1
*DGCR8*	Microprocessor Complex Subunit
*Dicer1*	Dicer 1, Ribonuclease III
DIV	Days in vitro
EB	embryoid body
*EFNA3*	Ephrin-A3
*EGF*	Epidermal Growth Factor
*EGFR*	Epidermal Growth Factor Receptor
EMT	epithelial-to-mesenchymal transition
*EphB2*	EPH Receptor B2
FACS	Fluorescence activated cell sorting
*FGF-2*	Fibroblast Growth Factor 2
*FOXO1*	Forkhead Box O1
GBM	glioblastoma multiforme
*GFAP*	Glial Fibrillary Acidic Protein
GSC	glioma stem cell
HCS	human cortical spheroid
hESC	human embryonic stem cell
*HES*	Hes Family BHLH Transcription Factor
HGG	high grade glioma
*HMGA*	High Mobility Group AT-Hook
*HEY1*	Hes Related Family BHLH Transcription Factor with YRPW Motif 1
hNPC	human neural progenitor cell
hNSC	human neural stem cell line
hPSC	human pluripotent stem cell
*ID4*	Inhibitor of Differentiation 4
*IMP*	Insulin-like Growth Factor 2 mRNA-Binding Protein
iPSC	induced Pluripotent Stem Cell
*ISCU*	Iron–Sulfur Cluster Scaffold Protein
*JAM-A*	Junctional Adhesion Molecule A
*KIP*	Cyclin Dependent Kinase Inhibitor
*KLF4*	Krüppel-like Factor 4
*LGALS3*	Galectin 3
*LHX2*	LIM Homeobox 2
*LIX1*	Limb and CNS Expressed 1
*MAP2*	Microtubule Associated Protein 2
*MAPK*	Mitogen-Activated Protein Kinase
MB	medulloblastoma
*MBNL1-3*	Muscleblind-like Splicing Regulator 1-3
*MEIS2*	Meis Homeobox 2
*MEK*	Mitogen-Activated Protein Kinase
*MIF*	Macrophage Migration Inhibitory Factor
*MIIP*	Migration and Invasion Inhibitory Protein
*MMP-12*	Matrix Metallopeptidase 12
*MNT*	MAX Network Transcriptional Repressor
MSC	melanocyte stem cell
*NOTCH*	Notch Receptor
*NRAS*	NRAS Proto-Oncogene, GTPase
*NR2F2*	Nuclear Receptor Subfamily 2 Group F Member 2
NSC	neural stem cell
*OCT4*	Octamer-Binding Transcription Factor 4
*ONECUT*	One Cut Homeobox
*PAX6*	Paired Box 6
*PDCD4*	Programmed Cell Death 4
*PDGFR*	Platelet-Derived Growth Factor Receptor
*PEG10*	Paternally Expressed 10
*PI3K*	Phosphoinositide-3-Kinase C
*PID1*	Phosphotyrosine Interaction Domain Containing 1
*PIM3*	Pim-3 Proto-Oncogene, Serine/Threonine Kinase
*PTBP1*	Polypyrimidine Tract Binding Protein 1
*PTEN*	Phosphatase and Tensin Homolog
RBPs	RNA-Binding Proteins
*RECK*	Reversion Inducing Cysteine Rich Protein with Kazal Motifs
*REST*	RE1-Silencing Transcription Factor
*RISC*	RNA induced silencing complex
*RSRC1*	Arginine and Serine Rich Coiled-Coil 1
*RTVP-1*	Related to Testis-Specific, Vespid, And Pathogenesis Proteins 1
*SART3*	Spliceosome Associated Factor 3, U4/U6 Recycling Protein
*SEC61G*	SEC61 Translocon Gamma Subunit
*SERTAD1*	SERTA Domain Containing 1
*SHH*	Sonic Hedgehog Signaling Molecule
*SIRT1*	Sirtuin 1
*SMAD*	SMAD Family Member
smNPC	small-molecule neural precursor cell
*SNAI2*	Snail Family Transcriptional Repressor 2
*SOX*	Sex Determining Region Y-Box
*S-TRAIL*	Secretable form of Tumor Necrosis Factor-Related Apoptosis Inducing Ligand
*TET3*	Tet Methylcytosine Dioxygenase 3
*TFAP2C/AP-2γ*	Transcription Factor AP-2 Gamma
*TGFβ*	Transforming Growth Factor β
*TLX*	T Cell Leukemia Homeobox
TMZ	temozolomide
*TPM1*	Tropomyosin 1
*TRBP*	TAR RNA binding protein
*TUBB3*	Tubulin Beta 3 Class III
*VEGF-A*	Vascular Endothelial Growth Factor A
WB	Western blot
*WNT*	Wingless-Related Integration Site
*YBX1*	Y-Box Binding Protein 1
